# Application of principles of exercise training in sub-acute and chronic stroke survivors: a systematic review

**DOI:** 10.1186/s12883-014-0167-2

**Published:** 2014-08-22

**Authors:** Bernadette C Ammann, Ruud H Knols, Pierrette Baschung, Rob A de Bie, Eling D de Bruin

**Affiliations:** 1 Directorate of Research and Education, Physiotherapy & Occupational Therapy Research, University Hospital Zurich, Gloriastrasse 25, Zurich, 8091, Switzerland; 2 Department of Epidemiology, CAPHRI School for Public Health and Primary Care, Maastricht University, Maastricht, 6200 MD, the Netherlands; 3 Department of Health, Institute of Physiotherapy, Zurich University of Applied Sciences, Technikumstrasse 9, Winterthur, 8401, Switzerland; 4 Department of Health Sciences and Technology, Institute of Human Movement Sciences and Sport, ETH Zurich, Wolfgang-Pauli-Str. 27, Zurich, 8093, Switzerland; 5 Centre for Evidence Based Physiotherapy, Maastricht University, Maastricht, 6200 MD, the Netherlands

**Keywords:** Stroke, Cerebrovascular accident, Aerobic exercise, Resistance exercise, Training principles, Training components

## Abstract

**Background:**

There is increasing evidence for the beneficial effects of exercise training in stroke survivors. In order to reach the desired training effects, exercise training principles must be considered as this ensures the prescription of adequate exercises at an adequate dose. Moreover, exercise training interventions must be designed in a way that maximizes patients’ adherence to the prescribed exercise regimen. The objectives of this systematic review were (1) to investigate whether training principles for physical exercise interventions are reported in RCTs for sub-acute and chronic stroke survivors, (2) to evaluate whether the RCTs reported the prescription of the FITT components of the exercise interventions as well as (3) patients’ adherence to this prescription, and (4) to assess the risk of bias of the included studies.

**Methods:**

We performed a systematic review of RCTs with exercise training as the primary intervention and muscular strength and/or endurance as primary outcomes. The Cochrane library’s risk of bias (ROB) tool was used to judge the methodological quality of RCTs.

**Results:**

Thirty-seven RCTs were included in this systematic review. Eighteen studies (48.7%) focused on aerobic, 8 (21.6%) on resistance and 11 (29.7%) on combined interventions of aerobic and resistive strength exercise. Twenty-nine studies (78.4%) included only chronic stroke survivors, 5 studies (13.5%) only sub-acute stroke survivors whilst 3 studies (8.1%) included both. In terms of principle of exercise training, 89% reported *specificity*, 75.7% *progression*, 48.7% *overload*, 37.8% *initial values*, 32.4% *reversibility* and 13.5% *diminishing returns*. One RCT described all principles of physical exercise training and 19 (51.4%) all FITT components. Patients’ adherence to exercise prescription was accounted for in 3 studies (8.1%). Failure to report blinding in patients and participants and failure to report allocation concealment were the most prevalent methodological shortcomings.

**Conclusions:**

Incomplete and inconsistent reporting of (1) training components, (2) underlying exercise training principles and (3) patient adherence together with (4) a broad variation in the methodological quality of the included RCTs limit both the utility and reproducibility of physical exercise programs in stroke patients.

## Background

Stroke is one of the leading causes of disability and death worldwide [1]. Due to demographic shifts in the global population, the number of affected people will increase, even with stable stroke incidence rates from approximately 1.1 million per year in 2000 to 1.5 million per year by 2025 [2],[3]. The difference by 2025 will be ± 150 000 stroke events when compared with stable rates [3]. Approximately 50-70% of persons with stroke regain functional independence, but 15-30% of the stroke survivors are left with permanent disability [4]. Disability - manifested by impairment of body function or body structure, activity limitation and/or participation restriction [5] - results in poor physical fitness, defined as “the ability to carry out daily tasks with vigor and alertness, without undue fatigue, and with ample energy to enjoy leisure-time pursuits and respond to emergencies” [6].

Physical fitness includes health-related (cardiorespiratory endurance, muscular endurance, muscular strength, flexibility and body composition) and skill-related components (agility, coordination, balance, speed, reaction time and power) [6]. All of these render the development of exercise training programs for stroke rehabilitation a complex enterprise. Although several exercise recommendations have been published [7]–[9], the complex interactions present in stroke rehabilitation preclude the definition of specific, detailed exercise prescriptions. Still, several systematic reviews and meta-analyses provide evidence that aerobic exercise and resistive strength training are beneficial to improve aerobic capacity, walking distance, muscular strength and physical function in stroke survivors without increasing pain or tone in the paretic limbs [10]–[17]. Thus, the available literature suggests that impaired physical fitness is partly responsible for the disability evident in stroke survivors.

When reporting the results of an exercise intervention, it is important that the precise principles of the exercise training are consistently and accurately reported [18],[19]. Recognised principles are *specificity, overload, progression, initial values, reversibility* and *diminishing returns* (Table 1) [18]–[20]. Their application in the design of an exercise intervention ensures that the dose and type of exercise is planned such that benefits for the recipient are maximized. Furthermore, it seems fair to assume that when principles of exercise training are applied to the development of exercise protocols, clinicians in practical settings can have greater confidence that non-significant research findings reflect deficiencies in exercise efficacy rather than deficiencies in exercise prescription [18].

**Table 1:** Exercise training principles

**Principle**	**Description**
**Specificity**	Exercising a certain body part or component of the body primarily develops that part: To become better at a particular exercise or skill, you must perform that exercise or skill.
**Overload**	A greater than normal stress or load on the body is required for training adaptation to take place. The body will adapt to this stimulus.
**Progression**	A gradual and systematic increase of the workload over a period of time will result in improvements in fitness without risk of injury.
**Initial values**	Improvement in the outcome of interest will be greatest in those with lower initial values. In other words, those with lowest level of fitness have greatest room for improvement.
**Reversibility**	Once a training stimulus is removed, fitness levels will eventually return to baseline (‘use it or lose it!’).
**Diminishing returns**	Refers to the decreasing expected degree of improvement in fitness as individuals become fit, thereby increasing the effort required for further improvements.

However, a perfectly planned intervention adopting all the principles of exercise training is almost useless when it is not reported in sufficient detail to permit intervention replication and results interpretation. Therefore, the prescription of the components of the exercise program and participants’ adherence to that exercise prescription should ideally be reported according to the so-called FITT components (*Frequency*, *Intensity*, *Time* and *Type* of exercise) (Table 2) [21]. FITT represents components of physical conditioning programs that determine effect on cardiorespiratory endurance, muscular strength and/or endurance and flexibility. Furthermore, participants’ adherence to each of the prescribed FITT components should be reported. Detailed reporting of the dose of exercise prescribed (and received) allows for an adequate interpretation of results – including possible dose–response effects – and provides information about the tolerability and safety of the intervention. Without detailed information on both the type and dose of exercise that is prescribed and actually received, developing optimally designed and dosed exercise prescriptions for a desired level of benefit (i.e., response) remains difficult, thus limiting the implementation of evidence-based training programs.

**Table 2:** FITT components applied to physical conditioning programs

**Frequency**	**Intensity**	**Time**	**Type**
The number of times an exercise or activity is performed generally expressed in sessions, episodes or bouts per week.	Refers to how much work is being performed or the magnitude of the effort required performing an activity or exercise.	The length or duration in which an activity or exercise is performed, usually expressed in minutes.	E.g. running/swimming for cardio respiratory endurance; free weights/resistance machines for muscular strength or endurance.

Well-designed randomized controlled trials (RCTs) provide the best evidence regarding the effectiveness of health care interventions. Trials with inadequate methodological approaches may overstate treatment effects and bias results. Critical appraisal of the quality of clinical trials is possible only if the design, execution and analysis of RCTs are described thoroughly and accurately [22],[23]. Thus, in order to properly interpret the results of an RCT, it is important to know the principles underlying the prescribed exercises, the FITT components of the intervention, the adherence to these components and the methodological quality of a trial.

The objectives of this systematic review were (1) to investigate whether training principles for physical exercise interventions are reported in RCTs for sub-acute and chronic stroke survivors, (2) to evaluate whether the RCTs reported the prescription of the FITT components of the exercise interventions as well as (3) patients’ adherence to this prescription, and (4) to assess the risk of bias of the included studies.

## Methods

### Search strategy

**Figure 1:**
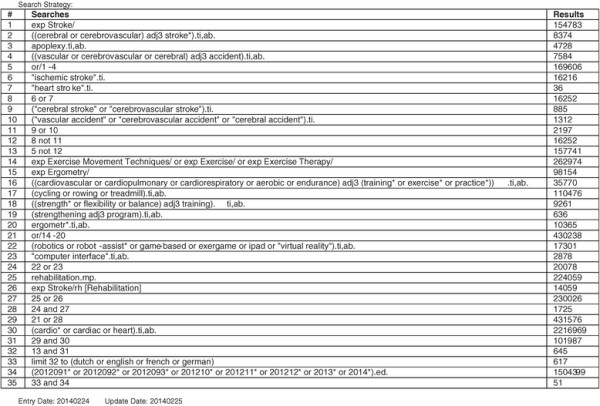
**Search history.** Example of search history from Ovid MEDLINE(R).

An electronic search strategy was developed and performed by a librarian of Zurich University, using the databases Medline, OvidSP, EMBASE, CINAHL, PsycInfo and the Cochrane Library (Figure 1). The search was restricted to English language literature from inception of the databases up to February 2014. Combinations of the following medical subject headings (MeSH) and free text words were used: stroke (cerebral stroke, vascular accident, brain vascular accident, apoplexy, cerebrovascular apoplexy, cerebrovascular stroke, cerebrovascular accident) and exercise (cardiovascular training, cardiopulmonary training, cardiorespiratory training, aerobic training, endurance training, exercise, endurance exercise, ergometry, resistive strength training, physical exercise principles, specificity, overload, progression, initial values, reversibility, diminishing returns).

### Eligibility criteria

Studies were eligible for inclusion if they: (1) were RCTs (2) evaluated the effects of aerobic physical exercise training or resistive strength training alone or in combination, (3) included adult (> 18 years) sub-acute or chronic stroke survivors, and (4) included aerobic capacity and/or aerobic endurance and/or muscle strength as primary outcome measurement. The sub-acute phase was defined as the range between 15 and 180 days after initial stroke, thus all RCTs with a mean or median within this time range were considered a sub-acute population. Chronic stroke was defined as the open-ended time period starting 180 days after initial stroke and characterised by generally slow or no clinical progress [24].

### Study selection

After removal of duplicates, the search results were screened for eligibility by two teams of two reviewers (BA/PB and RK/EDB), sharing the retrieved citations. In case of disagreement within one team, a reviewer from the other team served as referee.

### Data collection process

Using existing systematic reviews that evaluated the effects of exercise interventions in cancer patients [18],[19], BA created a purpose-designed data collection sheet to include information about each included RCT regarding (1) the reporting of exercise training principles, (2) the description of the exercise training components, (3) participants’ adherence to the training plan according the FITT components, and (4) further quality characteristics of the included studies (risk of bias (ROB)). Corresponding authors of selected trials were contacted by email to retrieve unpublished data. Three reviewers (BA, PB and RK) independently collected and rated these data.

### Rating of the reporting of exercise training principles

For each exercise principle, three rating categories were used: reported (+), not reported (−) and unclear or inconsistently reported (?).

### Rating of the description of exercise training components and participants’ adherence to the training plan

Corresponding to the rating of the training principles, we judged the description with (+) if the component of the exercise prescription was reported; (−) if the component was not reported and (?) if it was unclearly or inconsistently reported. Participants’ adherence to the exercise prescription was judged with (+) if the adherence of each component to the exercise prescription was reported; (−) if the adherence was not reported and (?) if it was unclearly or inconsistently reported.

### Rating of risk of bias in individual studies

Two reviewers (BA and PB) independently applied the Cochrane Collaboration’s tool for assessing ROB to assess the risk of over- or under-estimating the effects of an intervention [25]. The five bias domains (selection, performance, detection, attrition and reporting bias) were judged as: low ROB (+), unclear ROB (?) or high ROB (−). Rating (+) is unlikely to alter the results seriously, (?) raises some doubt about the results and (−) seriously weakens confidence in the results. With unclear information on an item, the score given was (?). The arbitration of a third reviewer (RK) was used in the event of any disagreement between the rating reviewers (BA and PB).

### Data synthesis

All extracted data from the RCTs were transformed into percentages. The percentage agreement and Cohen’s kappa were calculated and interpreted in accordance with Landis and Koch’s benchmarks for assessing the agreement between raters: poor (≤0), slight (between 0.0 and 0.20), fair (between 0.21 and 0.40), moderate (between 0.41 and 0.60), substantial (between 0.61 and 0.80), and almost perfect (between 0.81 and 1) [26]. The PRISMA statement was followed for reporting items of this systematic review [27],[28].

## Results

### Study selection

**Figure 2:**
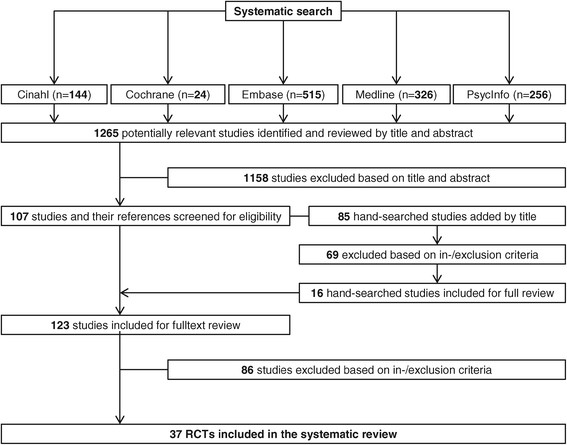
Study flow chart.

The systematic search provided a total of 1599 citations. After automatic de-duplication, 1265 hits remained. From this total, 1158 titles and abstracts failed to meet the inclusion criteria and were thus excluded. The remaining 107 abstracts were retrieved as full texts and screened together with their references. Sixteen additional studies were considered after manually scanning the reference lists of identified studies. After excluding another 86 studies based on in- and exclusion criteria, 37 studies were included in the final review (Figure 2).

### Study characteristics

From the 37 studies with a grand total of 2135 participants, 18 studies (48.7%) focused on aerobic exercises [29]–[46], 8 (21.6%) on resistance exercises [47]–[54] and 11 (29.7%) on combined interventions of aerobic and resistive strength exercise [55]–[65] (Additional file 1). Most of the included studies compared an experimental exercise intervention to usual care, or to an alternative intervention. Four RCTs included 3 or 4 training groups in their design [38],[53],[58],[59]. Twenty-nine studies (78.4%) included chronic stroke survivors [32]–[41],[43],[44],[46]–[54],[58]–[65], 5 (13.5%) included sub-acute stroke survivors [29]–[31],[55],[56] whilst 3 studies (8.1%) included both types of patients [42],[45],[57]. In the 18 studies evaluating aerobic exercise only (n = 1278) [29]–[46], treadmill training with or without body weight support was the most frequently used exercise intervention. In the 8 studies that focused on resistance training (n = 235) [47]–[54], circuit classes with progressive strength training constituted the most frequently used intervention. Of the 11 studies focusing on combined interventions of aerobic and resistive strength exercise (n = 622) [55]–[65], most focused on improvement of the lower extremity or both upper and lower extremities. No study focused on improvement of the upper extremity in the intervention group. The length of the exercise interventions ranged from 3 weeks [38] to 12 months [42],[45]. Sixteen studies (43.2%) described follow-up measurements [29]–[34],[36]–[38],[41],[47],[51],[54],[58],[61],[62] in which the follow-up period ranged from 2 months [51] to one year [32],[62].

### Application of exercise principles

One study described all six principles of exercise training [32], one described 5 out of 6 [47], 11 described 4 out of 6 [34],[36],[37],[40],[41],[43],[44],[46],[53],[58],[60] and 12 described three training principles [30],[35],[38],[39],[49],[51],[59],[61]–[64] (Additional file 2: Table S1a-c). The remaining 12 studies, representing 32.4% of the included studies, described just one [42],[48],[50],[57] or two [29],[31],[33],[45],[52],[54]–[56],[65] of these. With respect to the specific training principles, 33 (89.2%) of the RCTs reported *specificity*[30],[32],[34]–[56],[58]–[65], 28 (75.7%) described *progression*[32]–[41],[43],[44],[46],[47],[49],[51]–[53],[55],[56],[58]–[65], 18 (48.7%) described *overload*[32],[34]–[37],[39],[40],[43],[44],[46],[47],[49],[53],[58]–[60],[63],[64], 14 (37.8%) described *initial values*[29]–[32],[40],[41],[43]–[47],[53],[57],[60], 12 (32.4%) described *reversibility*[29],[31]–[34],[38],[41],[47],[51],[54],[61],[62] and 5 (13.5%) described *diminishing returns*[30],[32],[36],[37],[58]. Nineteen studies (51.4%) did not describe the *initial values* of their participants [34]–[37],[39],[42],[48]–[51],[55],[56],[58],[59],[61]–[65] and 14 (37.8%) did not describe the principle of *overload*[29]–[31],[33],[42],[48],[50],[51],[54]–[56],[61],[65]. Seven studies (18.9%) were unclear in describing *progression*[29]–[31],[42],[45],[48],[57], whilst 5 (13.5%) were unclear with respect to *overload*[38],[41],[52],[57],[62].

### Prescription of the exercise training

**Figure 3:**
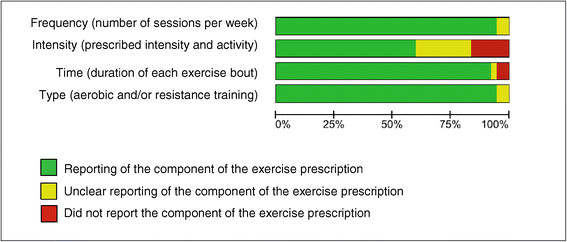
**Reporting of the FITT components.** Review authors’ judgments about the prescription of the training program according to the FITT components presented as percentages across all included studies.

The reporting of each FITT component of the exercise prescription is summarized in Figure 3. Details of the FITT component reporting of each study are presented in the Additional file 1. In general, *Frequency* was described in 35 studies (94.6%) [29]–[32],[34]–[41],[43]–[65], leaving 2 studies (5.4%) that were unclear in their reporting [33],[42]. Twenty-two studies (59.5%) reported training *Intensity*[32]–[37],[39]–[41],[43]–[47],[49],[50],[53],[58],[59],[61],[64],[65], 6 studies (16.2%) did not report this item [42],[51],[54]–[57] whilst 9 studies (24.3%) reported it unclearly [29]–[31],[38],[48],[52],[60],[62],[63]. Thirty-four studies (91.9%) reported the component *Time*[29]–[32],[34]–[48],[50]–[52],[54]–[65] and 35 RCTs (94.6%) reported *Type*[29]–[32],[34]–[56],[58]–[65] of physical exercise. Two studies (5.4%) did not report the *Time* dedicated to exercises [33],[49] whilst two studies (5.4%) unclearly reported the *Type* of each exercise bout [33],[57]. Nineteen studies (51.4%) described all FITT components [32],[34]–[37],[39]–[41],[43]–[47],[50],[58],[59],[61],[64],[65], 15 (40.5%) reported 3 of 4 FITT components [29]–[31],[38],[48],[49],[51]–[56],[60],[62],[63], two (5.4%) reported 2 out of 4 items [42],[57], and one (2.7%) reported one of the 4 items [33].

### Adherence to the exercise training

**Figure 4:**
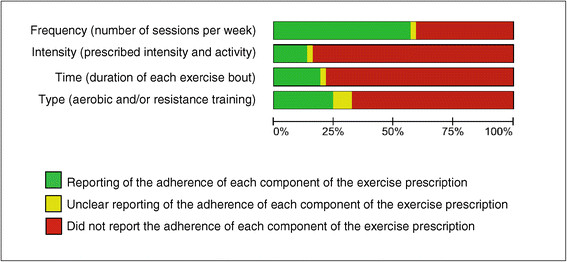
**Adherence to the FITT components.** Review authors’ judgments about the adherence of the study sample to the exercise prescription according to the FITT components presented as percentages across all included studies.

Adherence to each FITT component of the exercise prescription is summarized in Figure 4. Twenty-one studies (56.8%) reported the item *Frequency*[30],[32],[36],[37],[40]–[42],[46]–[52],[54]–[57],[61],[63],[65], 5 studies (13.5%) reported the item *Intensity* of the activity [32],[37],[46],[55],[58], 7 studies (18.9%) reported the item *Time* duration of each exercise bout [32],[37],[40]–[42],[55],[56] and 9 studies (24.3%) reported the item *Type* of exercise [32],[37],[40],[41],[46],[50],[52],[55],[61]. Three studies (8.1%) unclearly reported adherence to the *Type* of exercise [48],[54],[63] whilst 3 studies (each representing 2.7%) unclearly reported the items *Frequency*[60], *Intensity*[63] and *Time*[46] of the intervention. Fifteen (40.5%) studies did not report the item *Frequency*[29],[31],[33]–[35],[38],[39],[43]–[45],[53],[58],[59],[62],[64], 29 studies did not report the item *Time* (78.4%) [29]–[31],[33]–[36],[38],[39],[43]–[45],[47]–[54],[57]–[65], 25 studies did not report the *Type* of exercises completed (67.6%) [29]–[31],[33]–[36],[38],[39],[42]–[45],[47],[49],[51],[53],[56]–[60],[62],[64],[65] and the item *Intensity* was not reported in 31 studies (83.8%) [29]–[31],[33]–[36],[38]–[45],[47]–[54],[56],[57],[59]–[62],[64],[65]. Fifteen studies (40.5%) did not report any of the FITT components [29],[31],[33]–[35],[38],[39],[43]–[45],[53],[59],[60],[62],[64], 11 studies (29.7%) reported 1 out of 4 components [30],[36],[47]–[49],[51],[54],[57],[58],[63],[65] and 5 studies (13.5%) reported 2 out of 4 items [42],[50],[52],[56],[61]. Three studies (8.1%) reported all four FITT items [32],[37],[55].

### Methodological risk of bias

Methodological quality varied substantially across the studies (Additional file 3: Table S2a-c). All included studies had a low risk of selective reporting (reporting bias) [29]–[65], 19 (51.4%) had a low risk concerning the blinding of their outcome assessments (detection bias) [29],[31],[32],[34]–[36],[40],[42],[45],[46],[48]–[50],[52],[55],[57],[60],[61],[63], 21 studies (56.8%) had a low risk of incomplete outcome data (attrition bias) [32],[34],[35],[40]–[42],[46]–[50],[52],[54]–[57],[59],[61]–[63],[65], 19 studies (51.4%) had a low risk of selection bias concerning allocation concealment [32],[33],[35],[38],[40]–[46],[48],[50],[51],[56],[57],[61]–[63] and 22 studies (59.5%) showed a low risk of selection bias regarding random sequence generation [32],[33],[35]–[38],[40]–[42],[45],[46],[48],[51],[55]–[63]. With the exception of one study [40], the blinding of participants and personnel (performance bias) was a weak point in all studies: 30 studies (81.1%) had an unclear risk [29]–[39],[43],[44],[46],[48]–[60],[62],[64],[65] whilst the remaining 6 studies (16.2%) had a high risk of performance bias [41],[42],[45],[47],[61],[63].

The inter-rater agreement of the 3 rating categories “low, unclear, high ROB” of the quality assessment was substantial (Kappa: 0.71, SE of kappa: 0.04, 95%CI: 0.630-0.796). The number of observed agreements between the two raters was 83.3% of the judgments.

## Discussion

This systematic review evaluated the explicit reporting of the principles of (aerobic and/or resistance) exercise training in sub-acute and chronic stroke survivors. The results showed that these principles were inconsistently reported. This result impacts on the clinical reproducibility of trials, as clinicians cannot be confident whether non-significant findings are due to lack of efficacy or occur through limitations in treatment prescription. The risk of bias in the 37 studies depended on the bias domain being judged.

In this review, *specificity* and *progression* were the most frequently applied (i.e., explicitly reported) training principles, in 89.2% and 75.7% of reviewed studies respectively. Accordingly, most exercise trials clearly outlined training progression and designed their intervention to be specific to the target population, thus warranting reproducibility with respect to these training principles. In contrast, *initial values* and *overload* principles were not described in 37.8% and 48.7% of the reviewed RCTs respectively. Without knowing the baseline fitness levels of studied participants, it is difficult to generalize the findings to a clinical setting. Moreover, it is impossible to verify whether the provided exercise program was of adequate intensity, which hinders the interpretation of results. This situation is further aggravated by the fact that only 59.5% of the included studies reported the *Intensity* of their exercise training. Unfortunately, it is not always feasible to accurately measure the intensity of an exercise due to lack of equipment to monitor energy, work and/or power. In addition, a lack of knowledge of the exact effort (in terms of energy, work and/or power) that healthy subjects require to perform certain exercises [66] further complicates the dosing of exercises in patient populations. Only in studies where the mechanical output of physical activity can be controlled, such as by using cycle ergometry [35],[39] or leg press machines [49], is the required effort known. Yet even in light of these difficulties, it should always be possible to report the number of repetitions required in a certain exercise or the total time dedicated to exercise training. The latter was successfully described in 91.9% of the exercise trials included in this review. Furthermore, energy spent performing certain exercises might be assessed via proxy measures such as the Borg’s Rating of Perceived Exertion and Pain Scales [67]. Although perceived exertion is a subjective measure, it may nonetheless provide a fairly good estimate of the actual heart rate during physical activity and hence the intensity of that activity. Indeed, practitioners generally agree that perceived exertion ratings of 12 to 14 on the Borg Scale suggest a moderate level of intensity of physical activity [67].

There is evidence of a positive relationship between the time dedicated for therapy and therapy outcomes, indicating a positive relationship between dose and response [68],[69]. Lohse and colleagues [68] and Kwakkel et al. [69] both reported that the benefit of large increases in therapy volume is similar across a range of post-stroke times. That is, patients will benefit from an increase in therapy volume, regardless of whether their stroke occurred several months or several years ago. This finding also highlights the importance of exercise even after discharge from rehabilitation and reflects the training principle of *reversibility*: i.e. ‘use it or lose it’. Following the principle of *diminishing returns* – reported in only 13.5% of the studies – the effort required to achieve further improvements should increase over time. In line with the findings of the present systematic review, the principles of *diminishing returns* and *reversibility* were the least reported exercise principles in RCTs on physical training interventions in cancer survivors according to two recently published systematic reviews [18],[19]. A possible explanation is related to the fact that the assessment of these training principles requires follow-up measurements, which would increase research expenses and heighten the burden on patients participating in the study. However, assessment and reporting of both principles are crucial to determine the volume, frequency and intensity required in an exercise intervention to achieve durable long-term benefits for stroke patients [20].

Perhaps the most striking finding in this review was the discrepancy between the reporting of the FITT components in the exercise intervention (Figure 3) and the adherence to those components (Figure 4). To illustrate: 94.6% of the studies reported the FITT component *Type* yet only 24.3% of the studies reported adherence to this component. Similarly, *Time*, *Frequency* and *Intensity* was reported by 91.9%, 94.6% and 59.5% of the studies, respectively (Figure 3), yet adherence to these components was reported in only 18.9%, 56.8% and 13.5% of them (Figure 4). This general failure of reporting adherence with respect to the FITT components all but obscures this crucial aspect of an intervention [21] and hence prohibits important considerations that must be made before replicating an intervention in a clinical setting. There are several reasons why exercise programs might not be performed as prescribed, including patient-related factors (e.g. fatigue or depression [70],[71]; lack of motivation; stroke impairments [72],[73]), environmental factors (e.g. lack of transportation) or health concerns (e.g. fear of falls [74]). For clinicians and researchers alike, addressing these perceived barriers to exercise training is crucial both for successful rehabilitation and for provision of replicable exercise training programs. Promising first steps in breaking down these perceived barriers to training might be to remove costs for transportation or to integrate patients’ relatives in the rehabilitation process [75]. In support of the latter, it has been found that social support is an important motivator in achieving and maintaining physical fitness [72].

The most prevalent methodological shortcoming of the included studies was the absence of blinding of participants and personnel in 97.3% of the included RCTs. This is in line with findings of other systematic reviews of stroke exercise training [10],[16]. Such lack of blinding can cause overestimations of the treatment effect and therefore bias the study results. For example in a meta-epidemiological study by Wood et al. [76], estimates of treatment effects were exaggerated by 7% in non-blinded compared to blinded trials. Although blinding of participants and personnel may not always be feasible, it may still be possible to blind the outcome assessments. In this review, 19 RCTs (51.4%) had a low risk concerning this form of blinding (detection bias). Objectively assessed outcomes are less susceptible to bias than subjectively assessed ones [76]. Therefore, efforts to minimize other forms of bias are particularly important when objective measurements are not feasible. Allocation concealment was ambiguous in 48.6% of studies whilst 15 studies (40.5%) had a high or unclear risk of selection bias regarding random sequence generation. These findings are in line with those of other reviews [77]. Because concealment of allocation can lead to exaggeration of treatment effects [78], details of both sequence generation and concealment of allocation should always be clearly reported [22],[23].

Through improving cardiovascular fitness and muscle strength, disability after stroke may be reduced [16]. This is an important aspect of training since regaining function and independence are important goals for patients. The benefits of aerobic exercise might even be broader: Converging evidence suggests that aerobic exercise is a valuable intervention for improving brain function [79],[80] and promoting neuroplasticity by upregulation of neurotrophins [81]. Aerobic exercise programs after stroke have also been shown to improve blood pressure [82] and arterial function [83], as well as enhancing glucose regulation [84]. It is also highly plausible that exercise could be an effective treatment for fatigue [85], especially in combination with the treatment of the associated depressive symptoms of post-stroke fatigue (PSF) [86], even though it must be noted that there is insufficient evidence of an association between PSF and physical fitness to date [70]. Taken together, the total body of evidence clearly highlights the importance of maintaining physical fitness after stroke, as it greatly reduces the effort required to carry out daily tasks and therefore contributes to a more active and independent lifestyle [87]. Moreover, an optimal level of physical fitness decreases the risk of subsequent stroke, which is particularly significant in view of the fact that around 30% of stroke survivors will have recurrent stroke within their lifetimes, of which 18% will prove fatal [75]. Given the great restorative potential of achieving and maintaining physical fitness after stroke, the need for RCTs to properly report both exercise prescription and adherence to exercise prescription – which would allow full replication of positive findings in clinical settings – becomes particularly pressing.

### Call for transparency to facilitate evidence-based practice

In contemporary clinical practice, clinicians must ensure that the time allotted for therapy is used effectively and efficiently. To be successful, it is imperative that the goals of an exercise program be both reasonable and attainable [20],[88]. These aims are best achieved with a custom-made and individually tailored training program with variables that can be manipulated from workout to workout [21]. Such variables might be the choice, volume, intensity and order of exercise as well as the frequency and length of training and the length of rest periods. In order to achieve an optimum training effect, programs used in research should comply with and clearly report the exercise training principles [20] and FITT components [21]. This is expected to facilitate application of effective programs in clinical practice. However, the current state of the evidence still renders it difficult for practitioners to choose the optimal evidence-based training program for their individual patients. Only by reporting sufficient detail about volume and type of exercise actually performed by trial participants will more accurate interpretations of study outcomes and more appropriate translations of programs into non-research settings be permitted. A good starting point for future trials would be the American Heart Association (AHA) recommendation for stroke exercise training [89] (modified by Billinger et al. [75]). Such a detailed description of adherence, in combination with an equally detailed description of the developed program and the target population, would greatly facilitate reproduction of trials in clinical settings.

### Limitations of the review

To the best of our knowledge, this systematic review is the first to investigate the application of exercise training principles for sub-acute and chronic stroke patients. The review focuses on the reporting of intervention content rather than on the actual intervention outcomes. In striving to achieve a robust systematic review, we developed and documented the methods (e.g. a systematic search strategy and several worksheets for collecting and synthesizing the data) in advance. Due to the large number of existing trials on stroke rehabilitation, we decided to focus exclusively on RCTs to ensure high external validity. Nevertheless, some limitations remain. Firstly, we limited our search to English language peer-reviewed journal literature. Hence clearly there is a possibility that important RCTs published in other languages were missed. Secondly, due to the scope of the review, we did not perform meta-analyses of RCT results and hence cannot make recommendations concerning preferable exercise interventions for sub-acute and chronic stroke survivors. For training recommendations based on best available evidence we refer to the literature [75],[89]. Thirdly, although we included only RCTs in this review, the clinical and methodological diversity of the studies considered was still rather large. Finally, we exclusively focused on sub-acute and chronic stroke patients; thus our results cannot be generalized to acute patient groups. The reason for not including this patient population is twofold: First, there is no consensus in the literature as to how early physical activity should begin after a stroke [90] and second, information on how to influence and evaluate aerobic capacity in severely affected individuals is lacking [10].

## Conclusions

The present systematic review showed that RCTs on aerobic and/or resistance rehabilitation training in sub-acute and chronic stroke survivors incompletely and inconsistently reported the prescription of, and especially the adherence to, exercise prescriptions in investigated training programs. Allowing practitioners to use established training principles to guide the therapy process will help them to plan and implement their stroke therapy more efficiently and more effectively. Therefore it is of the utmost importance for researchers to plan and document investigated exercise interventions in as detailed a manner as possible. The consideration of all principles of exercise training and all FITT components in the development and reporting of exercise prescriptions is a course of action ideally fitted to this purpose.

## Competing interests

The authors declare that they have no competing interests.

## Authors’ contributions

BA conceived the methodology and carried out data collection, quality assessment, data analysis and manuscript writing. RK supervised progress, participating in methodology conception as well as quality assessment, data analysis and manuscript writing & revision. PB helped with data collection, quality assessment and data analysis. RDB contributed in manuscript writing. EDB initiated the study, supervised progress, helped with methodology conception, data collection and manuscript writing & revision. All authors read and approved the final manuscript.

## Additional files

## Supplementary Material

Additional file 1: Characteristics of included RCTs. Click here for file

Additional file 2: Table S10. Application of Principles of Exercise Training. a: Aerobic exercise only (n = 18). b: Resistance exercise only (n = 8). c: Aerobic and resistance exercises (n = 11). Click here for file

Additional file 3: Table S2. Rating of risk of bias in individual studies. a: Aerobic exercise only (n = 18). b: Resistance exercise only (n = 8). c: Aerobic and resistance exercises (n = 11). Click here for file
