# Supplementary material for: Application of principles of exercise training in sub-acute and chronic stroke survivors: a systematic review
Source: BMC Neurol. 2014 Aug 22;14:167. doi: 10.1186/s12883-014-0167-2 (PMC4236657; doi:10.1186/s12883-014-0167-2)
Supplement: Additional file 1: — Characteristics of included RCTs. [file s12883-014-0167-2-S1.pdf]

Additional file 1: Characteristics of included RCTs

	Study ID	Participants (Int/Con)	Intervention (Int)	Control (Con)	Primary outcomes (instrument)	Other outcomes (instrument)
**Aerobic exercise only (n=18)**						
**sub-acute stroke survivors**	Barbeau (2003) [29]	n=100<br>(50/50)<br>(LE/LE)	**F:** 4x/w for 6w<br>**I:** ur<br>**T:** 20min/d<br>**T:** TAEX with BWS +PT	**F:** 4x/w for 6w<br>**I:** ur<br>**T:** 20min/d<br>**T:** TAEX without BWS +PT	Walking endurance & speed (10MWT) **+**<br>Motor recovery (STREAM) **+**<br>Balance (BBS) **+**	
	Kosak (2000) [30]	n=56<br>(22/34)<br>(LE/LE)	**F:** 5d/w for duration of inpatient stay<br>**I:** ur<br>**T:** 45min/d<br>**T:** PBWSTT +PT of the same FIT	**F:** 5d/w for duration of inpatient stay<br>**I:** ur<br>**T:** 45min/d<br>**T:** ABAW+PT of the same FIT	Walking endurance (distance until fatigue) **0**<br>Walking speed (patients' average speed during 2min) **0**	
	Visintin (1998) [31]	n=100<br>(50/50)<br>(LE/LE)	**F:** 4x/w for 6w<br>**I:** ur<br>**T:** 20min/d<br>**T:** TAEX with BWS +PT	**F:** 4x/w for 6w<br>**I:** ur<br>**T:** 20min/d<br>**T:** TAEX without BWS +PT	Walking endurance (10MWT) **+**<br>Walking speed (middle 3m of a 10m-walkway) **+**<br>Motor recovery (STREAM) **+**<br>Balance (BBS) **+**	
**both**	Mayo (2013) [45]	n=87<br>(43/44)<br>(LE/LE)	**F:** daily for 12 months<br>**I:** 50-70% HRR<br>**T:** 15-30min/d<br>**T:** home ex. program with mobility ex. & brisk walking involving stationary cycling	**F:** daily for 12 months<br>**I:** 11-15 on Borg Scale (6-20 ratings)<br>**T:** 15-30min/d<br>**T:** home ex. program with mobility ex. & brisk walking	Walking capacity (6minWT) **0**	HRQoL (RAND-36) **0**<br>Balance (BBS) **0**<br>Physical function (SIS) **0**<br>Depression (GDS) **0**
	Dean (2012) [42]	n=151<br>(76/75)<br>(LE/UE)	**F:** at least 3x/w for 40w over 12 months<br>**I:** nr<br>**T:** 45-60min/d<br>**T:** home ex. program and weekly circuit-style group ex. class (progressive balance & strengthening ex., walking, stair climbing)	**F:** at least 3x/w for 40w over 12 months<br>**I:** nr<br>**T:** 45-60min/d<br>**T:** home ex. program and weekly circuit-style group ex. class to improve UE and cognitive functions (task-related strength & coordination training; sequencing tasks)	Walking capacity (6minWT) **+**<br>Walking speed (10MWT) **+**<br>Amount of falls (Fall calendars) **0**	Fall risk (SF-PPA) **0**<br>Physical activity (steps/d) (Digimax pedometer) **0**<br>HRQoL (SF-12) **0**<br>Community participation (AAP) **+**<br>Isometric knee muscle strength **0**<br>Activity (TUG) **0**
**chronic stroke survivors**	Bang (2013) [40]	n=30<br>(15/15)<br>(LE/LE)	**F:** 5x/w for 4 weeks<br>**I:** ½ of max overground walking speed, then increased<br>**T:** 30min/d plus 10min video<br>**T:** gait-related video, then treadmill training	**F:** 5x/w for 4 weeks<br>**I:** ½ of max overground walking speed, then increased<br>**T:** 30min/d plus 10min video<br>**T:** gait-unrelated video, then treadmill training	Walking endurance (6minWT) **+**<br>Dynamic balance test (TUG) **+**<br>Gait speed (10MWT) **+**<br>Walking capacity (max flexed knee angle in the swing phase (Dartfish motion analysis software)) **+**	
	Carda (2013) [41]	n=38<br>(19/19)<br>(LE/LE)	**F:** 5x/w for 6w<br>**I:** 80% of 6minWT speed, then progressively increased<br>**T:** 45min/d (PT) + 30min/d (treadmill training)	**F:** 5x/w for 6w<br>**I:** 80% of 6minWT speed, then progressively increased<br>**T:** 45min/d (PT) + 30min/d (treadmill training)	Walking endurance (6minWT) **+**	Gait speed (10MWT) **+**<br>Dynamic balance test (TUG) **+**

Additional file 1: Characteristics of included RCTs

			**T:** PT + treadmill training with 5% descending slope	**T:** PT + treadmill training with 5% ascending slope		
**chronic stroke survivors**	Jin<br>(2013) [43]	n=128<br><br>(65/63)<br><br>(LE/LE)	**F:** 5x/w for 12w<br>**I:** 50-70%HRR<br>**T:** 40min/d<br>**T:** progressive aerobic cycling	**F:** 5x/w for 12w<br>**I:** 20-30%HRR<br>**T:** 40min/d<br>**T:** conventional therapy (stretching and overground walking training)	Cardiovascular fitness ($V0_2$peak) **+**<br>HRR **+** (cycle ergometer)	Knee extension muscle strength (isokinetic dynamometer) **+**<br>Walking endurance (6minWT) **+**<br>Balance (BBS) **0**<br>Spasticity of LE (MASc) **0**
	Tang<br>(2013) [46]	n=50<br><br>(25/25)<br><br>(LE/LE)	**F:** 3x/w for 6 months<br>**I:** progressed from 40-80%HRR<br>**T:** 60min/d<br>**T:** high-intensity aerobic ex. (brisk level and inclined overground walking, cycle ergometry)	**F:** 3x/w for 6 months<br>**I:** < 40% HRR<br>**T:** 60min/d<br>**T:** low-intensity non-aerobic balance/flexibility program (stretching, weight bearing, balance ex.)	Cardiovascular fitness ($V0_2$peak) **0**	Arterial stiffness (pulse pressure in mm/Hg) **+**<br>Ambulatory capacity (6minWT) **0**<br>Hemodynamics & cardiac function (echocardiography; 2D-Doppler) **+**<br>Lipid, glucose & homocysteine levels **0**
	Globas<br>(2012) [32]	n=38<br><br>(20/18)<br><br>(LE/LE&UE)	**F:** 3d/w for 13w<br>**I:** 40-80% HRR<br>**T:** 30-50min/d<br>**T:** Progressive graded, high-intensity TAEX	**F:** 3d/w for 13w<br>**I:** 40-80% HRR<br>**T:** 30-50min/d<br>**T:** PT, then TAEX-cross	Peak exercise capacity ($V0_2$peak) **+**<br>Walking capacity (6minWT) **+**	Gait velocity (10MWT) **+**<br>Leg strength (5 chair-raise) **0**<br>Balance (BBS) **0**<br>Self-rated mobility (RMI) **+**<br>HRQoL (SF-12) **+**
	Jin<br>(2012) [44]	n=133<br><br>(68/65)<br><br>(LE/LE)	**F:** 5x/w for 8w<br>**I:** 50-70%HRR<br>**T:** 40min/d (plus 30min+10min)<br>**T:** progressive aerobic cycling with lower limb weights on the paretic limb (plus balance exercise and stretching)	**F:** 5x/w for 8w<br>**I:** 20-30% HRR<br>**T:** 40min/d (plus 30min+10min)<br>**T:** low intensity overground walking (plus balance exercise and stretching)	Cardiorespiratory fitness ($V0_2$peak) **+**<br>Walking endurance (6minWT) **+**<br>Mobility (RMI) **0**	Muscle strength (isokinetic dynamometer) **+**<br>Balance (BBS) **0**<br>Spasticity of LE (MASc) **0**
	Moore<br>(2010) [33]	n=20<br><br>(10/10)<br><br>(LE/LE)	**F:** 2-5d/w for 4w for PT and LT<br>**I:** 0-85% HRR or Borg 17/ LT session<br>**T:** nr<br>**T:** Progressive TAEX with BWS<br>4w PT- 4w LT- 4w delay period	**F:** 2-5d/w for 4w for PT and LT<br>**I:** 0-85%HRR or Borg 17/ LT session<br>**T:** nr<br>**T:** Progressive TAEX with BWS<br>4w PT- 4w delay period- 4w LT	Self-selected **0** and fastest velocity **+** (Gait MAt II)<br>Walking endurance (12MWT) **0**<br>Physical fitness (O_2 cost **+**; peak treadmill speed **+**; VO_2 **0**)<br>Mobility (TUG **0**; StepWatch **+**)<br>Balance (BBS) **0**	
	Quaney<br>(2009) [34]	n=38<br><br>(19/19)<br><br>(LE/LE&UE)	**F:** 3d/w for 8w<br>**I:** 40-70% HRR<br>**T:** 45min/d<br>**T:** Progressive, resistive aerobic ex. on a stationary bike	**F:** 3d/w for 8w<br>**I:** nr<br>**T:** 45min/d<br>**T:** Stretching ex. at home	Vo_2 max (metabolic stress test) **+**<br>Mobility (TUG) **+**<br>Motor learning (SRTT) for response time **+** / for random sequence response time **0**<br>Balance (BBS) **0**<br>Sensorimotor function (FMMA) **0**	

Additional file 1: Characteristics of included RCTs

					Conditional learning ability (PGFM) **+**<br>Rule learning (WCST) **0**<br>Selective attention (Stroop task21) **0**<br>Visual search ability & working memory (Trail-Making Task) **0**	
**chronic stroke survivors**	Lennon (2008) [35]	n=48<br>(24/24)<br>(LE&UE / LE&UE)	**F:** 2d/w for 10w<br>**I:** 50-60% HRR<br>**T:** 30min/d<br>**T:** Cycle ergometer ex.	**F:** nr<br>**I:** nr<br>**T:** nr<br>**T:** Usual care (excluding aerobic ex.)	Fitness testing ($V0_2$ **+**; RPE **0**; peak wattage (Nm) **+**; CRS **+**; resting heart rate (bpm) **0**; blood pressure **0**; BMI **0**; fasting lipids **0**)<br>Daily activities (FAI) **0**<br>Depression (HADS) **0**	
	Luft (2008) [36]	n=113<br>(37/34)<br><br>(LE/LE)	**F:** 3d/w for 6 month<br>**I:** 40-60% HRR<br>**T:** 40min/d<br>**T:** Progressive TAEX<br>*Subset (N=15) for fMRI testing*	**F:** 3d/w for 6 month<br>**I:** nr<br>**T:** 40min/d<br>**T:** Stretching<br>*Subset (N=17) for fMRI testing*	Cardiorespiratory fitness ($V0_2$peak) **+**<br>Gait velocity (10MWT) **+**<br>Walking ability & endurance (6minWT) **0**	*fMRI* **+**
	Macko (2005) [37]	n=61<br>(32/29)<br>(LE/LE)	**F:** 3d/w for 6 month<br>**I:** 40-70% HRR<br>**T:** 40min/d<br>**T:** Progressive TAEX	**F:** 3d/w for 6 month<br>**I:** Treadmill at 30-40% HRR<br>**T:** 35min / 5min<br>**T:** Stretching **/** Treadmill walking	Cardiorespiratory fitness ($V0_2$peak L/min) **+**<br>Economy of gait ($V0_2$ mL/kg/min) **0**<br>Ambulatory performance measures (10MWT **0**; 6minWT **+**)<br>Functional Mobility (RMI **0**; WIQ **+**)	
	Peurala (2005) [38]	n=45<br><br>(15/15/15)<br><br>(LE/LE/LE)	**F:** 5d/w for 3w & additional PT 5d/w for 3w for all groups<br>**I:** ur<br>**T:** 20min/d & additional PT 55min/d for all groups<br>**T:** G1: Gait trainer ex. with FES<br>G2: Gait trainer ex. without FES<br>G3: Practicing walking		Walking speed (10MWT) **0** & endurance (6minWT) **0**<br>Postural sway (force plate) **0**<br>Spasticity of LE (MASc) **+** in the ankle (only in G3) but **0** in the knee and hip (all groups)<br>Muscle force (scale 0-5) **+** for the ankle dorsiflexors (only in G1) and **+** for the hip flexors (only in G2) but **0** for the knee extensors (all groups)<br>Motor ability (MMAS) **0**<br>Functional independence (FIM) **0**	
	Potempa (1995) [39]	n=42<br><br>(19/23)<br><br>(LE/LE&UE)	**F:** 3d/w for 10w<br>**I:** 20-94 watts<br>**T:** 30min/d<br>**T:** Progressive cycle ergometer	**F:** 3d/w for 10w<br>**I:** nr<br>**T:** 30min/d<br>**T:** PROM ex.	Physical fitness (HR at rest and during ex. (RR interval) **0**; blood pressure (sphygmomanometer) for the systolic **+** but for the diastolic blood pressure during ex. **0**; metabolic parameters VO_2 **+**; VCO_2 **+**; VE **+** and RER **0** (MGCESTS); workload (ergometer) **+**; exercise time (ergometer) **+**)<br>Sensorimotor function (FMMA) **0**	

Additional file 1: Characteristics of included RCTs

Resistance exercise only (n=8)						
	**Study ID**	**Participants (Int/Con)**	**Intervention (Int)**	**Control (Con)**	**Primary outcomes (instrument)**	**Other outcomes (instrument)**
**chronic stroke survivors**	Clark (2013) [52]	n=35<br>(18/17)<br>(LE/LE)	**F:** 3x/w for 5w (plus 3w)<br>**I:** 3-4x 10 reps (nr)<br>**T:** 90min/d (90min/d)<br>**T:** ECC-PRT of the paretic leg (followed by PBWSTT)	**F:** 3x/w for 5w (plus 3w)<br>**I:** 3-4x 10 reps (nr)<br>**T:** 90min/d (90min/d)<br>**T:** CON-PRT of the paretic leg (followed by PBWSTT)	CON **0** and ECC **0** muscle strength quadriceps (isokinetic dynamometer)<br>Muscle activity of the (RF+VM) **+**, (ST+BF) **0** (EMG)<br>Walking speed (motion analysis system) **0**	
	Lee (2013) [53]	n=33<br>(11/11/11)<br>(LE/LE/nr)	**F:** 5x/w for 6w<br>**I:** 3 sets of 70% of 1RM<br>**T:** ur<br>**T:** G1: PRT with CKC ex. on a leg press<br>G2: PRT with OKC ex. on a leg press<br>G3: routine activities, no regular exercise program		LE muscle activity (in %) of the RF **+**, BF **+**, GC **+** + and TA **+** +<br>(EMG)<br>Balance (Good Balance System platform: (A-P) **+** + & (M-L) **+** + sway velocities (mm/s))<br>+ (more significant changes in favor of G1 in post hoc analysis)	
	Waldman (2013) [54]	n=24<br>(12/12)<br>(LE/LE)	**F:** 3x/w for 6w<br>**I:** nr<br>**T:** 60min/d<br>**T:** active movement training for strengthening ankle muscles plus passive stretching for ROM therapy through a portable robotic device	**F:** 3x/w for 6w<br>**I:** nr<br>**T:** 60min/d<br>**T:** stretching plantar flexors and AROM exercises for ankle mobility and strength	Isometric ankle muscle strength (DF **+** & PF **0**) / ankle AROM (DF **0** & PF **0**) and PROM (DF **+**) (robotic device)<br>LE muscle endurance (6minWT) **0**<br>LE movement (STREAM) **+**<br>Gait evaluations (F-SCAN; in-shoe plantar pressure mapping system) **+**<br>Balance ability (BBS) **0**<br>Plantar flexor spasticity (MASc) **+**	
	Flansbjer (2008) [47]	n=24<br>(15/9)<br>(LE/LE&UE)	**F:** 2d/w for 10w<br>**I:** 6-8 reps in 2 sets, 80% 1RM<br>**T:** 90min/d with 6min PRT<br>**T:** PRT of the knee muscles	**F:** nr<br>**I:** nr<br>**T:** nr<br>**T:** Usual daily activities	Muscle strength (dynamic **+**& isokinetic **+**; MASc **0**)<br>Gait performance (TUG **+**; Fast gait speed **0**; 6minWT **0**)<br>Perceived participation (SIS) **+**	
	Yang (2006) [48]	n=48<br>(24/24)<br>(LE/LE&UE)	**F:** 3d/w for 4w<br>**I:** ur<br>**T:** 30min/d<br>**T:** ex. for LE strengthening (circuit class)	**F:** nr<br>**I:** nr<br>**T:** nr<br>**T:** Usual care	LE muscle strength (dynamometer) **+**<br>LE muscle endurance (6minWT) **+**<br>Gait velocity, cadence & stride length (GAITRite) **+**<br>Mobility (TUG) **+**<br>Balance (step test) **+**	
	Ouellette (2004) [49]	n=42<br>(21/21)<br>(LE/LE&UE)	**F:** 3d/w for 12w<br>**I:** 3 sets,8-10 reps/set,70% 1RM<br>**T:** nr<br>**T:** Supervised high-intensity PRT with 4 ex.	**F:** 3d/w for 12w<br>**I:** nr<br>**T:** nr<br>**T:** Bilateral ROM ex. + UE stretching	LE muscle strength (1RM Leg Press **+**; knee extension ex.**+**; DF-ex. in the paretic **+** & in the non-paretic limb **0**; PF-ex. **+**; peak muscle power Leg Press **0**; knee extension ex. **+**)<br>LE muscle endurance (6minWT) **0**<br>Stair-climb time (10-stair flight) **0**<br>Repeated chair-rise time **0**<br>Gait velocity (10MWT) **0**<br>Function & disability (LLFDI) **0**	Self-perceived ability for 5 functional tasks (ESES) **0**<br>Self-reported functional outcome (PF10) **0**<br>Depression (GDS) **0**

Additional file 1: Characteristics of included RCTs

**chronic stroke survivors**	Kim (2001) [50]	n=20<br>(10/10)<br><br>(LE/LE)	**F:** 3d/w for 6w<br>**I:** 3 sets, 10 reps/set at RM<br>**T:** 45min/d<br>**T:** max concentric isokinetic strength training	**F:** 3d/w for 6w<br>**I:** nr<br>**T:** 45min/d<br>**T:** PROM of paretic LE	Isokinetic strength of LE (dynamometer) **0**<br>Walking speed (level-walking and stair-walking) **0**<br>HRQoL (SF-36) **0**<br>Mental health (MSC) **0**
	Dean (2000) [51]	n=9<br><br>(5/4)<br><br>(LE/UE)	**F:** 3d/w for 4w<br>**I:** nr<br>**T:** 60min/d<br>**T:** Task-related strength training (circuit of 10 workstations)	**F:** 3d/w for 4w<br>**I:** nr<br>**T:** 60min/d<br>**T:** Practiced UE tasks (circuit of workstations & ex.)	Walking speed (10MWT) **+** and endurance (6minWT) **+**<br>LE function; force production (TUG) **+**<br>Balance (step test) **+**

Additional file 1: Characteristics of included RCTs

Aerobic and resistance exercises (n=11)						
	**Study ID**	**Participants (Int/Con)**	**Intervention (Int)**	**Control (Con)**	**Primary outcomes (instrument)**	**Other outcomes (instrument)**
**sub-acute stroke survivors**	Duncan (2003) [55]	n=92<br><br>(44/48)<br><br>(LE&UE / LE&UE)	**F:** 3d/w for 12w<br>**I:** nr<br>**T:** 90min/d<br>**T:** Supervised in-home strength, endurance and balance program	**F:** nr<br>**I:** nr<br>**T:** nr<br>**T:** Usual care + completing intervention log	Muscular strength (dynamometer and ankle & knee isometric peak torque) **0**<br>Cardiorespiratory endurance (peak aerobic capacity) **+**<br>UE-function (WMFT) **0**<br>LE & UE motor control (Fugl-Meyer) **0**<br>Mobility (10MWT **0**, 6minWT **+**)<br>Balance (BBS) **+**	
	Duncan (1998) [56]	n=20<br>(10/10)<br><br>(LE&UE / LE&UE)	**F:** 3d/w for 12w<br>**I:** nr<br>**T:** 90min/d<br>**T:** Supervised in-home strength, endurance and balance program	**F:** nr<br>**I:** nr<br>**T:** nr<br>**T:** Usual care + completing intervention log	Gait speed (10MWT) **+** & walking distance (6minWT) **0**<br>Motor recovery LE **+** and UE **0** (FMMA)<br>ADL (BI of ADL) **0** and (LS-IADL) **0**<br>HRQoL (SF-36) **0**<br>Balance (BBS) **0**<br>Upper extremity function (Jebsen test) **0**	
**both**	Salbach (2004) [57]	n= 91<br><br>(44/47)<br><br>(LE/UE)	**F:** 3d/w for 6w<br>**I:** nr<br>**T:** 60min/d<br>**T:** 10 functional tasks to strengthen the LE & to improve walking balance, speed &distance	**F:** 3d/w for 6w<br>**I:** nr<br>**T:** nr<br>**T:** Seated UE activities	Walking capacity (6minWT) **+**	Comfortable and max pace (5MWT) **+**<br>Mobility (TUG) **0**<br>Balance (BBS) **0**
**chronic stroke survivors**	Lee (2010) [58]	n=48<br><br>(12/12/12/12)<br><br>(LE/LE/ LE/LE)	**F:** 3d/w for 12w for all groups<br>**I:** PRT: 2 sets, 8 reps/set, 50-80% 1RM<br>Cycling: 50-80% HRR<br>**T:** 30min/d for all groups<br>**T:** G1: aerobic cycling + sham PRT<br>G2: sham cycling + PRT<br>G3: aerobic cycling + PRT<br>G4: sham cycling + sham PRT		Max muscle force (pneumatic resistance machines; fixed dynamometer) **+ 0**<br>Max muscle power (peak force, velocity) **+ 0**<br>Muscle endurance (as many repetitions as possible) **+ 0**<br><br>Strength/PRT<br>Aerobic/cycling	
	Lee (2008) [59]	n=52<br><br>(13/13/14/12)<br><br>(LE/LE/ LE/LE)	**F:** 3d/w for 12w for all groups<br>**I:** PRT: 2 sets, 8 reps/set, 50-80% 1 RM<br>Cycling: 50-80% HRR<br>**T:** 30min/d for all groups<br>**T:** G1: aerobic cycling + sham PRT<br>G2: sham cycling + PRT<br>G3: aerobic cycling + PRT<br>G4: sham cycling + sham PRT		Mobility (10MWT, 6minWT) **0 0**<br>Stair climbing test & power **+ 0**<br><br>Strength/PRT<br>Aerobic/cycling	Max muscle force (pneumatic resistance machines; fixed dynamometer) **+ 0**<br>Muscle endurance (as many repetitions as possible) **+ 0**<br>Cardiorespiratory fitness **+** ($V0_2$peak, PO peak **+**, peak HR)<br>HRQoL (SF-36) **+ 0**
	Pang (2008) [60]	n=63<br><br>(32/31)	**F:** 3d/w for 19w<br>**I:** ur<br>**T:** 60min/d<br>**T:** FAME: cardiorespiratory	**F:** 3d/w for 19w<br>**I:** nr<br>**T:** 1h/d<br>**T:** Seated UE program, no	Walking capacity (6minWT) **+**<br>$V0_2$ max (Excalibur cycle ergometer) **+**<br>Muscle strength (dynamometer) in the paretic **+** & in the non-paretic knee **0**	

Additional file 1: Characteristics of included RCTs

		(LE/UE)	fitness, mobility, balance, LE strengthening	aerobic ex., no LE strengthening, no balance	Balance (ABCS **0**; BBS **0**)
chronic stroke survivors	Mead (2007) [61]	n=66<br>(32/34)<br><br>(LE&UE / LE&UE)	**F:** 3d/w for 12w<br>**I:** Endurance: at least Borg 13, PRT: 4-15 reps per ex.<br>**T:** 75min/d<br>**T:** Progressive endurance training (cycle ergometer) & PRT	**F:** 3d/w for 12w<br>**I:** nr<br>**T:** 75min/d<br>**T:** Relaxation	Physical fitness (V0$_2$ mL/kg **+**; NPR **0**)<br>Mobility (RMI **0**; reach **0**; sit-to-stand **0**; elderly mobility score **0**; TUG **+**)<br>ADL (FIM **0**; NE-ADL **0**)<br>HRQoL (SF-36) **+**<br>Depression (HADS) **0**
	Olney (2006) [62]	n=74<br><br>(38/36)<br><br>(LE/LE)	**F:** 3d/w for 10w<br>**I:** ur<br>**T:** 90min/d<br>**T:** Supervised ex. sessions: graded walking and/or cycling program+ strength training	**F:** 3d/w for 10w<br>**I:** nr<br>**T:** 90min/d<br>**T:** 1w supervised & 9w unsupervised ex. Sessions at home, (same sessions as intervention group)	Walking endurance (6minWT) **0**<br>Strength of LE muscles (dynamometer) **0**<br>Oxygen cost (PCI) **0**<br>Activity (HAP) **+**<br>Physical and mental health (SF36) **0**
	Pang (2005) [63]	n=63<br><br>(32/31)<br><br>(LE/UE)	**F:** 3d/w for 19w<br>**I:** ur<br>**T:** 60min/d<br>**T:** FAME: mobility, cardiorespiratory fitness, balance, LE strengthening	**F:** 3d/w for 19w<br>**I:** ur<br>**T:** 60min/d<br>**T:** Seated UE program, no aerobic ex., no LE strengthening, no balance	VO$_2$ max (Excalibur cycle ergometer) **+**<br>Walking endurance (6minWT) **+**<br>Muscle strength (dynamometer) in the paretic **+** & in the non-paretic knee **0**<br>Activity and participation (PASIPD) **0**<br>Femoral neck bone mineral density (dual-energy x-ray absorptiometry) in the paretic **+** & in the non-paretic leg **0**<br>Balance (BBS) **0**
	Carr (2003) [64]	n=40<br><br>(nr/nr)<br><br>(LE&UE/ LE&UE)	**F:** 3d/w for 16w<br>**I:** Endurance: 40-70% of max wattage, Strength: 2sets, 10 reps/set<br>**T:** 20-40min/d + strength ex.<br>**T:** Aerobic & strength training (same aerobic program & progressively increasing strength-training)	**F:** 3d/w for 16w<br>**I:** 40-70% of max wattage<br>**T:** 20-40min/d<br>**T:** Aerobic training only (UE&LE body ergometer)	V0$_2$max (stress test) **+**<br>Muscle strength (Biodex strength assessment): knee flexion, knee extension **0**, shoulder extension&flexion **+**<br>Blood lipid panel (cholesterol **+**, glucose **0**, lipids **0**)
	Teixeira-Salmela (1999) [65]	n=13<br><br>(6/7)<br><br>(LE/LE)	**F:** 3d/w for 10w<br>**I:** Aerobic ex.: 50-70% HRR, Strength: 3sets, 10 reps/set, 50-80% 1RM<br>**T:** 60-90min/d<br>**T:** Graded walking& stepping or cycling& LE muscle strengthening	**F:** nr<br>**I:** nr<br>**T:** nr<br>**T:** No intervention for 10w, then the same 10-week program as intervention group	Comfortable gait speed (middle 22meters of 30meters) **+**<br>Activity per day (AAS) **+**<br>Quality of life (NHP) **+**<br>Comfortable stair-climbing speed **+**<br>Physical activity (HAP) **+**<br>LE muscle strength (dynamometer) **+**<br>LE spasticity of quadriceps (pendulum test) **0** & ankle PF (controlled resistance to passive stretch) **0**

Additional file 1: Characteristics of included RCTs

Legend: + = significant between-group difference / 0 = non-significant difference between groups / both in favor of intervention group.

AAP = Adelaide Activities Profile, ABAW = aggressive bracing assisted walking, ABCS = Activity-Specific Balance Confidence Scale, ADL = Activities of Daily Living, (A-L) = antero-posterior, BBS = Berg Balance Scale, BF = biceps femoris, BI = Barthel Index, BMI = Body Mass Index, BWS = body weight support, BWSTT = body weight supported treadmill training, CKC = closed kinetic chain, CMSA = Chedoke-McMaster Stroke Assessment, CON = concentric, CRS = Cardiac Risk Score, d = days, DF = dorsiflexion, ECC = eccentric, EMG = electromyography, ESES = Ewart's Self Efficacy Scale, ex. = exercise, FAI = Frenchay Activities Index, FAME = Fitness and Mobility Exercises, FIM = Functional Independence Measure, FMMA = Fugl-Meyer Motor Assessment, fMRI = functional Magnet Resonance Imaging, GC = gastrocnemius, GDS = Geriatric Depression Scale, HADS = Hospital Anxiety and Depression Scale, HAP = Human Activity Profile, Hfmax = maximal heart rate, HR = heart rate, HRR = Heart Rate Reserve, HRQoL = Health Related Quality of Life, LE = lower extremity, LLFDI = Late Life Function and Disability Instrument, LS-IADL = Lawton Scale of Instrumental Activities of Daily Living, LT = locomotor training, MAS = Motor Assessment Scale, MASc = Modified Ashworth Scale, max = maximum, min = minutes, minWT = minute walking test, (M-L) = medio-lateral, MSC = Mental Health Component Summary Score, MWT = meter walking test, NE-ADL = Nottingham Extended Activities of Daily Living, NPR = Nottingham Power Rig, nr = not reported, OKC = open kinetic chain, PADS = Physical Activity and Disability Scale, PASIPD = Physical Activity for Individuals with Physical Disability, PBWSTT = Partial body weight supported treadmill training, PCI = Physiological Cost Index, PF = plantar flexion, PF10 = Physical Functioning Subscale, PGIC = Patient Global Impression of Change, PNF = Proprioceptive Neuromuscular Facilitation Patterns, PO = power output, PPA = Physiological Profile Assessment, PROM = Passive Range of Motion, PRT = Progressive resistance training, PT = Physical Therapy, reps = repetitions, RF = rectus femoris, (1)RM = (One) Repetition Maximum, RMI = Rivermead Mobility Index, RPE = Borg Rate of Perceived Exertion, SF = Short Form, SF36 = 36-item Short-Form Health Survey, SIS = Stroke Impact Scale, ST = semitendinosus, STREAM = Stroke Rehabilitation Assessment of Movement, TA = tibialis anterior, TAEX = Aerobic Treadmill Exercise, TUG = Timed up and go test, UE = upper extremity, ur = unclear reporting, VM = vastus medialis, $V0_2$ = oxygen consumption, w = weeks, WIQ = Walking Impairment Questionnaire, WMFT = Wolf Motor Function Test, FES = Functional Electric Stimulation.
