# Supplementary material for: Application of principles of exercise training in sub-acute and chronic stroke survivors: a systematic review
Source: BMC Neurol. 2014 Aug 22;14:167. doi: 10.1186/s12883-014-0167-2 (PMC4236657; doi:10.1186/s12883-014-0167-2)
Supplement: Additional file 2: Table S10. — Application of Principles of Exercise Training. a: Aerobic exercise only (n = 18). b: Resistance exercise only (n = 8). c: Aerobic and resistance exercises (n = 11). [file s12883-014-0167-2-S2.pdf]

Additional file 2: Table S1 Application of Principles of Exercise Training

a: Aerobic exercise only (n=18)

	Study ID	Specificity	Progression	Overload	Initial values	Reversibility	Diminishing returns
sub-acute stroke survivors	Barbeau (2003) [29]	?	?	-	+	+	-
	Kosak (2000) [30]	+	?	-	+	-	+
	Visintin (1998) [31]	?	?	-	+	+	-
both	Mayo (2013) [45]	+	?	-	+	-	-
	Dean (2012) [42]	+	?	-	-	-	-
chronic stroke survivors	Bang (2013) [40]	+	+	+	+	-	-
	Carda (2013) [41]	+	+	?	+	+	-
	Jin (2013) [43]	+	+	+	+	-	-
	Tang (2013) [46]	+	+	+	+	-	-
	Globas (2012) [32]	+	+	+	+	+	+
	Jin (2012) [44]	+	+	+	+	-	-
	Moore (2010) [33]	?	+	-	?	+	-
	Quaney (2009) [34]	+	+	+	-	+	-
	Lennon (2008) [35]	+	+	+	-	-	-
	Luft (2008) [36]	+	+	+	-	-	+
	Macko (2005) [37]	+	+	+	-	-	+
	Peurala (2005) [38]	+	+	?	?	+	-
	Potempa (1995) [39]	+	+	+	-	-	-

Additional file 2: Table S1 Application of Principles of Exercise Training

b: Resistance exercise only (n=8)

	Study ID	Specificity	Progression	Overload	Initial values	Reversibility	Diminishing returns
chronic stroke survivors	Clark (2013) [52]	+	+	?	?	-	?
	Lee (2013) [53]	+	+	+	+	-	-
	Waldman (2013) [54]	+	-	-	?	+	-
	Flansbjer (2008) [47]	+	+	+	+	+	-
	Yang (2006) [48]	+	?	-	-	-	-
	Ouellette (2004) [49]	+	+	+	-	-	-
	Kim (2001) [50]	+	-	-	-	-	-
	Dean (2000) [51]	+	+	-	-	+	-

Additional file 2: Table S1 Application of Principles of Exercise Training

c: Aerobic and resistance exercises (n=11)

	Study ID	Specificity	Progression	Overload	Initial values	Reversibility	Diminishing returns
sub-acute stroke survivors	Duncan (2003) [55]	+	+	-	-	-	-
	Duncan (1998) [56]	+	+	-	-	-	-
both	Salbach (2004) [57]	?	?	?	+	-	-
chronic stroke survivors	Lee (2010) [58]	+	+	+	-	-	+
	Lee (2008) [59]	+	+	+	-	-	-
	Pang (2008) [60]	+	+	+	+	-	-
	Mead (2007) [61]	+	+	-	-	+	-
	Olney (2006) [62]	+	+	?	-	+	-
	Pang (2005) [63]	+	+	+	-	-	-
	Carr (2003) [64]	+	+	+	-	-	-
	Teixeira-Salmela (1999) [65]	+	+	-	-	-	-

Legend:

+	Application of principle
-	Principle not reported
?	Unclear use or inconsistently applied
