# Supplementary material for: Application of principles of exercise training in sub-acute and chronic stroke survivors: a systematic review
Source: BMC Neurol. 2014 Aug 22;14:167. doi: 10.1186/s12883-014-0167-2 (PMC4236657; doi:10.1186/s12883-014-0167-2)
Supplement: Additional file 3: Table S2. — Rating of risk of bias in individual studies. a: Aerobic exercise only (n = 18). b: Resistance exercise only (n = 8). c: Aerobic and resistance exercises (n = 11). [file s12883-014-0167-2-S3.pdf]

Additional file 3: Table S2 Rating of risk of bias in individual studies

a: Aerobic exercise only (n=18)

	**Study ID**	**Random sequence generation (selection bias)**	**Allocation concealment (selection bias)**	**Blinding of participants and personel (performance bias)**	**Blinding of outcome assessment (detection bias)**	**Incomplete outcome data (attrition bias)**	**Selective reporting (reporting bias)**
**sub-acute stroke survivors**	Barbeau (2003) [29]	?	?	?	+	-	+
	Kosak (2000) [30]	?	?	?	?	-	+
	Visintin (1998) [31]	?	?	?	+	-	+
**both**	Mayo (2013) [45]	+	+	-	+	-	+
	Dean (2012) [42]	+	+	-	+	+	+
**chronic stroke survivors**	Bang (2013) [40]	+	+	+	+	+	+
	Carda (2013) [41]	+	+	-	-	+	+
	Jin (2013) [43]	?	+	?	?	?	+
	Tang (2013) [46]	+	+	?	+	+	+
	Globas (2012) [32]	+	+	?	+	+	+
	Jin (2012) [44]	?	+	?	?	?	+
	Moore (2010) [33]	+	+	?	?	-	+
	Quaney (2009) [34]	?	?	?	+	+	+
	Lennon (2008) [35]	+	+	?	+	+	+
	Luft (2008) [36]	+	?	?	+	-	+
	Macko (2005) [37]	+	?	?	-	-	+
	Peurala (2005) [38]	+	+	?	?	-	+
	Potempa (1995) [39]	?	?	?	?	?	+

Additional file 3: Table S2 Rating of risk of bias in individual studies

b: Resistance exercise only (n=8)

	**Study ID**	**Random sequence generation (selection bias)**	**Allocation concealment (selection bias)**	**Blinding of participants and personnel (performance bias)**	**Blinding of outcome assessment (detection bias)**	**Incomplete outcome data (attrition bias)**	**Selective reporting (reporting bias)**
**chronic stroke survivors**	Clark (2013) [52]	?	?	?	+	+	+
	Lee (2013) [53]	-	?	?	?	?	+
	Waldman (2013) [54]	-	?	?	?	+	+
	Flansbjer (2008) [47]	?	?	-	-	+	+
	Yang (2006) [48]	+	+	?	+	+	+
	Ouellette (2004) [49]	?	?	?	+	+	+
	Kim (2001) [50]	?	+	?	+	+	+
	Dean (2000) [51]	+	+	?	?	-	+

Additional file 3: Table S2 Rating of risk of bias in individual studies

c: Aerobic and resistance exercises (n=11)

	**Study ID**	**Random sequence generation (selection bias)**	**Allocation concealment (selection bias)**	**Blinding of participants and personnel (performance bias)**	**Blinding of outcome assessment (detection bias)**	**Incomplete outcome data (attrition bias)**	**Selective reporting (reporting bias)**
sub-acute stroke survivors	Duncan (2003) [55]	+	?	?	+	+	+
	Duncan (1998) [56]	+	+	?	?	+	+
both	Salbach (2004) [57]	+	+	?	+	+	+
chronic stroke survivors	Lee (2010) [58]	+	?	?	-	-	+
	Lee (2008) [59]	+	?	?	-	+	+
	Pang (2008) [60]	+	?	?	+	-	+
	Mead (2007) [61]	+	+	-	+	+	+
	Olney (2006) [62]	+	+	?	-	+	+
	Pang (2005) [63]	+	+	-	+	+	+
	Carr (2003) [64]	?	?	?	?	-	+
	Teixeira-Salmela (1999) [65]	?	?	?	?	+	+

Legend:

+	Low risk
-	High risk
?	Unclear risk

Additional file 3: Table S2 Rating of risk of bias in individual studies
